# No evidence for size-assortative mating in the wild despite mutual mate choice in sex-role-reversed pipefishes

**DOI:** 10.1002/ece3.907

**Published:** 2013-12-11

**Authors:** Kenyon B Mobley, Maria Abou Chakra, Adam G Jones

**Affiliations:** 1 Max Planck Institute of Evolutionary BiologyAugust-Thienemann Str. 2, Plön, 24306, Germany; 2 Department of Biology, Texas A&M University3258 TAMU, College Station, Texas, 77843, USA

**Keywords:** Assortative mating, body size, mark–recapture, mate choice, sexual selection, Syngnathidae

## Abstract

Size-assortative mating is a nonrandom association of body size between members of mating pairs and is expected to be common in species with mutual preferences for body size. In this study, we investigated whether there is direct evidence for size-assortative mating in two species of pipefishes, *Syngnathus floridae* and *S. typhle*, that share the characteristics of male pregnancy, sex-role reversal, and a polygynandrous mating system. We take advantage of microsatellite-based “genetic-capture” techniques to match wild-caught females with female genotypes reconstructed from broods of pregnant males and use these data to explore patterns of size-assortative mating in these species. We also develop a simulation model to explore how positive, negative, and antagonistic preferences of each sex for body size affect size-assortative mating. Contrary to expectations, we were unable to find any evidence of size-assortative mating in either species at different geographic locations or at different sampling times. Furthermore, two traits that potentially confer a fitness advantage in terms of reproductive success, female mating order and number of eggs transferred per female, do not affect pairing patterns in the wild. Results from model simulations demonstrate that strong mating preferences are unlikely to explain the observed patterns of mating in the studied populations. Our study shows that individual mating preferences, as ascertained by laboratory-based mating trials, can be decoupled from realized patterns of mating in the wild, and therefore, field studies are also necessary to determine actual patterns of mate choice in nature. We conclude that this disconnect between preferences and assortative mating is likely due to ecological constraints and multiple mating that may limit mate choice in natural populations.

## Introduction

Assortative mating is nonrandom mating based on similarity (Burley 1983; Jiang et al. 2013) and may arise via sexual selection when either one or both partners evolve preferences for mates with trait values similar to their own (Crespi 1989; Arnqvist et al. 1996; Härdling and Kokko 2005). Organisms use a wide range of phenotypic traits for assortative mating, including body size (Arnqvist et al. 1996), ornamentation (Andersson et al. 1998; Hancox et al. 2010), and major histocompatibility complex genotype (Milinski 2006). Such nonrandom patterns of mating should be especially common in natural populations when traits used in mate choice confer a fitness advantage or reflect variation in genotypic quality (Arnqvist 2011). Alternatively, assortative mating may occur in the absence of mate choice as a consequence of various constraints on mating such as temporal or spatial segregation of mating types, intrasexual competition, and intersexual conflict (Crespi 1989; Arnqvist et al. 1996; Arnqvist 2011; Jiang et al. 2013). Negative, or disassortative mating (i.e., preferentially mating with mates of dissimilar phenotypes), can also occur although it is rarely documented in nature (Jiang et al. 2013).

Size-assortative mating, which often arises from a preference for larger mates, may evolve through mutual mate choice for body size or by strong mating preferences in one sex, in which case the sex with stronger preferences sets the upper limit on the strength of the association (McNamara and Collins 1990; Arnqvist et al. 1996). Patterns of size-assortative pairing are found in a wide diversity of taxa, including, for instance, invertebrates (Arnqvist et al. 1996; Johnson 1999; Bollache and Cézilly 2004), fish (McKaye 1986; Baldauf et al. 2009), reptiles (Shine et al. 2001), and birds (Helfenstein et al. 2004; for taxonomic review, see Jiang et al. 2013). Mating with individuals of larger size can confer a fitness or fecundity advantage and can evolve through mate choice for fitter partners or offspring (Arnqvist et al. 1996; Arnqvist 2011). Larger individuals may also gain access to preferred mates through greater competitive ability or a reduction in costs associated with contest competition (Arnqvist et al. 1996; Härdling and Kokko 2005). Size-assortative mating can also be a means to resolve sexual conflict for mating preferences and may play a role in the maintenance of sexually antagonistic genetic variation (South et al. 2009; Arnqvist 2011; Thünken et al. 2012).

In some instances, strong mating preferences for individuals of large body size may not translate into assortative mating particularly if individuals of the preferred range of body sizes are unavailable or reluctant to mate. For example, the reluctance of larger individuals to pair with smaller individuals may come at a high fitness cost in terms of the number of offspring. Therefore, preferences may be relaxed if other larger mates are not available. Numerous ecological factors also must play an important role in the manifestation of size-assortative mating. For example, environmentally induced variation in mate quality, mate availability, and resource competition may all potentially influence the strength of mating preferences at a given time (Crespi 1989; Arnqvist et al. 1996; Bollache and Cézilly 2004).

In this study, we test the hypothesis that size-assortative mating occurs in natural populations of two species of pipefish, *Syngnathus typhle* (*L*.) and *Syngnathus floridae* (Jordan & Gilbert). Species in the genus *Syngnathus* have exclusive paternal care with embryos brooded in a specialized pouch, are sex-role reversed in relation to the strength of sexual selection, and have a polygynandrous mating system where both males and females mate multiply (Berglund et al. 1988; Jones and Avise 1997b; Jones et al. 1999; Mobley and Jones 2009; Mobley et al. 2011b). Mate-choice experiments conducted in *S. typhle* demonstrate a preference for larger body size in individuals of the opposite sex (Berglund et al. 1986, 2005; Berglund and Rosenqvist 1993; Berglund 1994). The strength of mate choice also depends heavily on the operational sex ratio (OSR, the ratio of adult males to females ready to mate) experienced during pairing (Berglund 1994). Thus, studies suggest that *S. typhle* should mate size-assortatively in the wild based on strong mutual preference for larger body size (Berglund et al. 1986). It is currently unknown whether *S. floridae* also show mutual preferences for larger body size, but laboratory studies on mate choice suggest that males, but possibly not females, prefer mates of larger body size in a Texas population (S. Scobell, pers. comm. 2013).

To investigate assortative mating by body size in these two species, we sampled two geographically distinct populations of *S. floridae* and the same *S. typhle* population between two different years. Each collection was sampled intensively and used a microsatellite-based parentage analysis to identify the mates of pregnant males collected from the field. This method allows for a direct comparison of body size among males and the females with which they mated in the wild. We also explored the effects of mating order and number of eggs transferred per female on size-assortative mating patterns in these species. Finally, we constructed a simulation-based model to investigate whether size-assortative mating should be expected in pipefish. The motivation for such a model was to simulate the strength and directionality of preferences by males and females that may generate the observed patterns of pairing in nature. Therefore, our heuristic model simulated both positive and negative size-assortative mating, and a range of the strength of preferences for body size from weak (or no) preferences to strong preferences. Male and female preferences were also allowed to vary independently such that all potential preference possibilities were explored, including positive assortative mating, negative assortative mating, and antagonistic mating preferences (males and females have different mating preferences).

Contrary to our expectations, we found no evidence of size-assortative mating in *S. floridae* and *S. typhle* in our field collections, and no evidence that either order of mating or number of eggs transferred by females affected pairing patterns in the wild. Further, our simulations also demonstrate that strong preferences for body size are unlikely to explain natural patterns of mate pairing in our populations. Our study shows that while preferences for traits may exist through laboratory-based mate-choice trials, these preferences may not manifest into assortative mating patterns in nature.

## Methods

### Empirical methods

Adult *S. typhle* were collected from Trinnhålet bay (58°14′23.32″N, 11°22′44.86″E) on the island of Gåsö on the west coast of Sweden during the months of May and June in 2005 as well as June of 2006. Fish were collected from a single continuous, shallow (1–6 m) eelgrass bed using a beam trawl with a 2-mm mesh towed from a boat. Adult *S. floridae* were collected from Morehead City, North Carolina, (34°43′20.54″N, 76°45′24.98″W) in June of 2004 and Aransas Pass, Texas (27°52′50.16″N, 97°6′6.84″W), in July of 2006 from shallow seagrass beds using a 2-mm mesh hand-drawn seine net. All individuals of both species were sexed and measured for body length (standard length, tip of rostrum to the caudal peduncle) and were either fin clipped (nonpregnant males and females) or sacrificed (pregnant males) for genetic analysis. We calculated the adult sex ratio as the total number of adult males divided by the total number of adults [males/(males + females)] at the time of collection and the operational sex ratio as the number of nonpregnant males (i.e., males that had no eggs in the brood pouch) divided by the sum of nonpregnant males and adult females.

A Gentra PureGene™ cell and tissue kit (Qiagen, Hilden, Germany) was used to extract DNA from adult fin tissue. Brood pouches of pregnant males were dissected, and individual embryos were placed in a 5% Chelex/Proteinase K digestion (Miller and Kapuscinski 1996). Adult tissue and every fourth embryo of *S. typhle* were genotyped using three microsatellite loci *Typh04*, *Typh16*, and *Typh18* (Jones et al. 1999). Adult tissue and embryos of *S. floridae* were genotyped with three microsatellite loci, *Micro11.1*, *Micro22.3,* and *Micro25.22* (Jones and Avise 1997a) using protocols previously reported (Mobley and Jones 2007, 2009). All microsatellite fragment analyses were performed on an ABI Prism® 3730 DNA Analyzer, and resulting fragments were scored using ABI Prism®GeneMapper™ software (Applied Biosystems, Foster City, CA).

Maternal genotypes were reconstructed from progeny arrays using GERUD2.0 (Jones 2005), and the cumulative probability of identity (*P*_ID_) of field-caught females was estimated from microsatellite data using LOCUSEATER2.4 (Hoyle et al. 2005). Field-caught females were matched to reconstructed maternal genotypes using Microsatellite Toolkit 3.1 (Park 2001). Female recapture rate was calculated as the number of reconstructed maternal genotypes matched to field-caught females and expressed as a percentage. A modified Lincoln–Petersen method of mark–recapture was used to estimate local female population size based on the number of reconstructed female genotypes present (Jones and Avise 1997b; Mobley and Jones 2007, 2009).

Analysis of variance (ANOVA) models were employed to test for significant differences in body size between the sexes from population estimates between years (*S. typhle*) or geographic locations (*S. floridae*). We used regression analyses to investigate the body size relationships between males and females that mated with each other. We then constructed general linear mixed models (GLMMs) to investigate the relationship of female body size (response variable) to male body size using mating order and percentage of eggs contributed by each female as covariates. Previous work has shown that males accept disproportionately more eggs from the first female in several species of pipefishes (Berglund et al. 1988; Partridge et al. 2009; Paczolt and Jones 2010). Therefore, we divided females into two groups: (1) first females and (2) all females that mated after the first female, and then used female mating order as an ordinal covariate in the GLMM models. Additionally, larger females generally provide more and larger eggs per copulation (Berglund and Rosenqvist 2003; Partridge et al. 2009; Mobley et al. 2011a), so we used the percentage of eggs contributed per female as a covariate. Because several females can mate with the same male, we included the unique identification of males (Male ID) as a random effect in the model to account for the nonindependence of male body size in these cases. All ANOVAs were first run with all interactions, and GLMM models were run with all second-level interactions; all nonsignificant interactions were removed systematically starting at the highest order. All statistical analyses were conducted using PASW18.0 (SPSS Inc. Chicago, IL).

### Theoretical methods

We developed a general computational model to simulate various mate pairing patterns under different mate-choice regimes. We considered a single trait, body size (T), and we posited that individuals have a specific body size preference strength (P). These individual preference strengths influence the distribution of mating pairs in a population, which results in a change in the overall patterns of mating at the population level. Applying individual preferences in a computational model can establish which preference range best explains the patterns of matings observed in the natural pipefish populations sampled in this study.

Simulations are conducted using two heuristics: positive and negative. The positive heuristic states that individuals choose mates with body sizes within a range around their own (T_i_ ± T_i_ * P, where T_i_ is the focal individual's body size, and P is the preference). The negative heuristic states that individuals choose mates with body size contrasting with their own body size (T_min_ + (T_max_ − T_i_) ± T_i_ * P, where T_min_ and T_max_ refer to the smallest and largest body sizes in each population). These heuristics explore preference conditions and do not address how individual mates are chosen. Also, these heuristics are not perfect “mirror models”; we simply chose the most parsimonious and simplest conditions that encompass a diverse range of preferences from strong to weak. We then conducted large-scale simulations for three possible scenarios: (1) positive assortative mating, both mates choosing based on a positive heuristic; (2) negative assortative mating, both mates choosing based on a negative heuristic; (3) antagonistic mating preferences, one mate choosing based on a negative heuristic and the other choosing based on the positive one. For more information, refer to the interactive .cdf model SuppProgram in the supporting information.

We used a rigorous algorithm exploring all possible mating pairs (P_1_, P_2_ allowed to vary independently) chosen from a real number distribution ranging from 0 to 1.5 in increments of ±0.025 for each scenario. For instance, under the positive heuristic, a focal individual could display a preference for mates of exactly the same size (P = 0, high preference) or may have a wide preference range, mating with individuals 1.5 times larger or smaller than the focal individual's body size (P = 1.5, no preference). We chose P = 1.5 as the theoretical preference limit so that all individuals in each population could be sampled under no preference. The simulation was replicated for each scenario, and natural population of pipefish sampled. We sampled 100 mating pairs from the population; this process was repeated for a total of 10,000 iterations for each collection of pipefish. We then regressed male size on female size and compared these simulated results with regressions from *S. typhle* and *S. floridae* natural populations. We assume that multiple matings are possible such that an individual can be sampled more than once.

Comparisons between collected populations and simulated populations were conducted using an algorithm (supporting information) that measured the accuracies and distance between the linear regressions from the two data sets. From such a comparison, we can quantify the corresponding preferences under the three preference scenarios.

All computer models and associated statistical analyses were programmed using the *Mathematica* 8.0 platform (Wolfram Research Inc., Champaign, IL, USA). All fish handling was carried out under the auspices of Animal Use Protocol #2004-227 issued by Texas A&M University.

## Results

### Assortative mating in natural populations of pipefishes

Data concerning the number of individuals collected in each population, adult and operational sex ratios, population sizes, and body length distributions are found in Table 1. All populations of pipefishes sampled had adult sex ratios that did not differ from equality (chi-squared test, P > 0.05) and operational sex ratios that were significantly female-biased, a typical pattern for sex-role-reversed species (Table 1). Female population size was generally lower in *S. floridae* than in *S. typhle* (Table 1).

**Table 1:** Summary statistics for *Syngnathus typhle* and *Syngnathus floridae*. Listed for each population is the number of adult males and females (*n*), adult sex ratio (ASR), operational sex ratio (OSR), mean male mating success, mean male reproductive success, female population size, number of male–female-matched mating pairs, number of females matched to males using parentage analysis as a function of the total number of females captured (female recapture), and population mean and range of body size of males and females

	*S. typhle*		*S. floridae*	
		
	Gåsö (2005)	Gåsö (2006)	North Carolina	Texas
Males (*n*)	67	55	33	30
Females (*n*)	84	35	49	32
ASR^1^	0.48	0.61	0.40	0.48
OSR^2^	0.03	0.08	0.13	0.06
Mean male mating success	3.3 ± 0.3	3.6 ± 0.4	1.4 ± 0.2	1.8 ± 0.1
Mean male reproductive success	82.0 ± 7.1	87.5 ± 5.9	157.6 ± 20.8	330.5 ± 30.8
Female pop. size (95% C. I.)	295 (83–377)	205 (70–276)	76 (32–140)	67 (19–87)
Matched mating pairs	18	28	21	28
Female recapture (%)	13.1	40.0	30.6	40.6
Mean male body size	182.0 ± 3.2	164.8 ± 4.1	124.2 ± 2.2	149.9 ± 3.9
(range)	(122–267)	(117–253)	(102–144)	(117–192)
Mean female body size	192.5 ± 4.5	192.2 ± 6.5	128.3 ± 5.1	136.5 ± 3.0
(range)	(106–273)	(115–270)	(99–162)	(104–160)

1 ASR = males/(males + females).

2 OSR = nonpregnant males/(nonpregnant males + females).

Female *S. typhle* were significantly larger than males in 2006 (ANOVA: *F*_1,88_ = 13.928, *P* < 0.001) but not in 2005 (ANOVA: *F*_1,149_ = 3.323, *P* = 0.070), and overall body size of males and females differed between the 2 years (2-way ANOVA year: *F*_1,238_ = 4.165, *P* = 0.042). No sexual size dimorphism in body size was detected in North Carolina (ANOVA: *F*_1,77_ = 1.796, *P* = 0.184). However, Texas males were significantly larger than females (ANOVA: *F*_1,50_ = 7.505, *P* = 0.008). Individuals of both sexes hailing from the Texas population were significantly larger than their North Carolinian counterparts (2way ANOVA local * sex: *F*_1,137_ = 9.788, P = 0.002; Table 1).

In all populations, we found a high number of reconstructed female genotypes that matched an exact three locus genotype of females that were caught in the field. Cumulative probabilities of identity (*P*_ID_) based on microsatellite markers for females were very low, ranging from 4.6 × 10^−7^ to 7.5 × 10^−7^ for *S. typhle* and from 1.5 × 10^−5^ to 8.5 × 10^−6^ for *S. floridae*, so the probability that two females would share the same genotype was virtually nil in both species. The number of female reconstructed genotypes matched to females caught in the field ranged from 13.1 to 40.6% of the total number of females sampled for each population (Table 1). Many females mated with more than one male within each collection yielding a high proportion of matched male–female pairs (Mobley 2007; Mobley and Jones 2009, 2013; Table 1).

**Figure 1:**
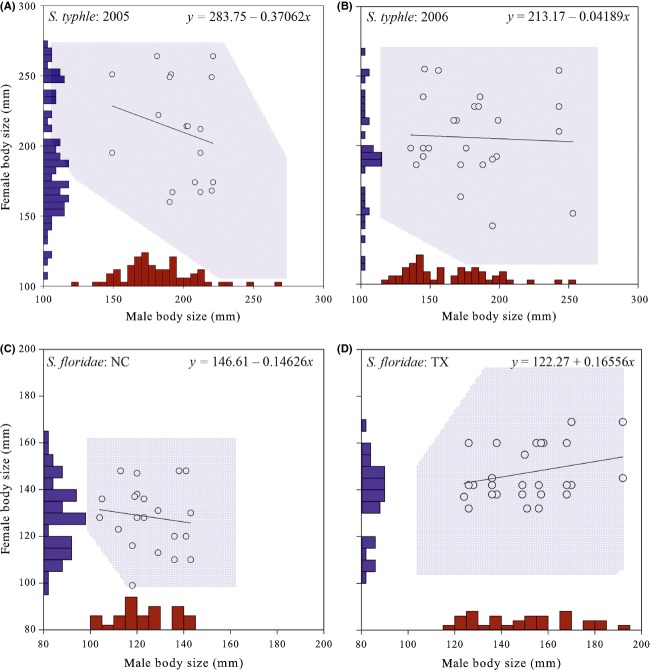
The relationship of female body size and male body size matched using microsatellite-based parental reconstruction in *S. typhle* collected from Gåsö, Sweden, in (A) 2005 and (B) 2006 and *S. floridae* from (C) North Carolina (NC) and (D) Texas (TX). Distribution of body size are shown in 5-mm increments for females (blue) and males (red) for all adults sampled in each population. Regression lines and equations are provided to show the direction of the relationship. No regression is significant (*P* > 0.05). Shaded regions represent the best-fit preference ranges for the negative (A, B, C) or positive (D) heuristic models (see text for model descriptions).

Based on parentage analysis, we found no evidence of a significant relationship between female body size and male body size in either *S. typhle* or *S. floridae*. In three of the four populations, we found a trend toward negative size-assortative mating (Gåsö 2005: *r* = −0.221, df = 16, *P* = 0.379; Gåsö 2006: *r* = −0.052, df = 26, *P* = 0.793, North Carolina: *r* = −0.128, df = 19, *P* = 0.581; Fig. 1). Only in Texas did we see a potential for positive size-assortative mating (*r* = 0.275, df = 26, *P* = 0.156; Fig. 1). Results of the two GLMMs also showed that neither the order of mating nor the percent of eggs contributed by females influence size-assortative mating patterns in either species (Table 2).

**Table 2:** General linear mixed model (GLMM) analysis testing the relationship between female body size (response variable) and male body size. Location and year are categorical factors, order of mating is an ordinal factor, and male body size and percent of eggs contributed are covariates. Male ID was used as a random factor in each model

	df	*F*	*P*
*Syngnathus typhle*			
Year	1,35.9	0.822	0.372
Male body size	1,22.2	1.005	0.327
Order	1,40.0	0.158	0.693
Percentage of eggs contributed	1,40.7	1.088	0.303
*Syngnathus floridae*			
Location	1,26.2	7.248	0.012
Male body size	1,25.3	1.039	0.318
Order of mating	1,25.8	2.532	0.124
Percentage of eggs contributed	1,41.3	2.289	0.138

### Model results

**Figure 2:**
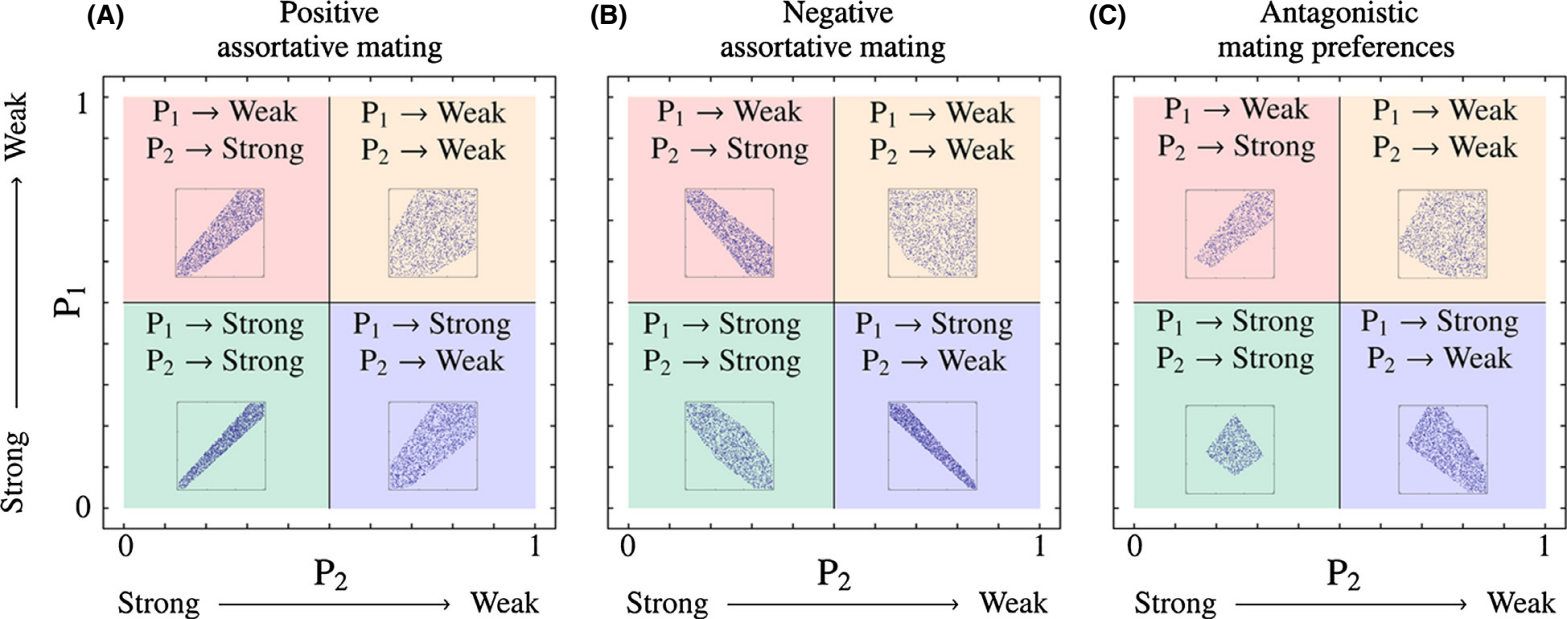
Overview of model simulations for the three preference scenarios. The preference by mate 1 (P_1_) and mate 2 (P_2_) are independent and are defined by either the positive heuristic model (T_i_ ± T_i_ * P) or the negative heuristic model (T_min_ + T_max_ − T_i_ ± T_i_ * P). Each panel is divided into four regions depicting how the strength of P_1_ and P_2_ influences the overall mating pairs in the population. Three possible scenarios are explored: (A) positive assortative mating; both P_1_ and P_2_ use the positive heuristic. (B) Negative assortative mating; both P_1_ and P_2_ use the negative heuristic. (C) Antagonistic mating preferences; P_1_ uses the negative heuristic and P_2_ uses the positive heuristic.

Our model demonstrated that the strength of assortative mating observed depends on the individual who has the greater preference strength (P) in each population. For example, Fig. 2A shows that small P_1_ and P_2_ values (strong preferences) result in strong assortative mating based on body size. As P_1_ increases (preferences become weaker) and the corresponding individuals become less choosy (wider preference range), the sex with the stronger preference determines the strength of the relationship between male and female body size. This effect is symmetrical when P_1_ and P_2_ are identical. Similarly, the choosier sex sets limits on the range of pairing patterns for the negative model (Fig. 2B). Under the antagonistic mating preference scenario (Fig. 2C), the slope of the relationship can vary based on the strength of preferences within each sex. If the strength of preferences is equal under this scenario, the slope of the relationship between male and female body size will be zero. If both sexes display strong antagonistic preferences, a small range of mate pairs can be sampled and only the individuals near the population mean body size will be able to mate. However, if preferences are asymmetrical, then the choosier mate again will set the upper limit on the strength of the interaction and will determine whether the slope is positive or negative.

**Figure 3:**
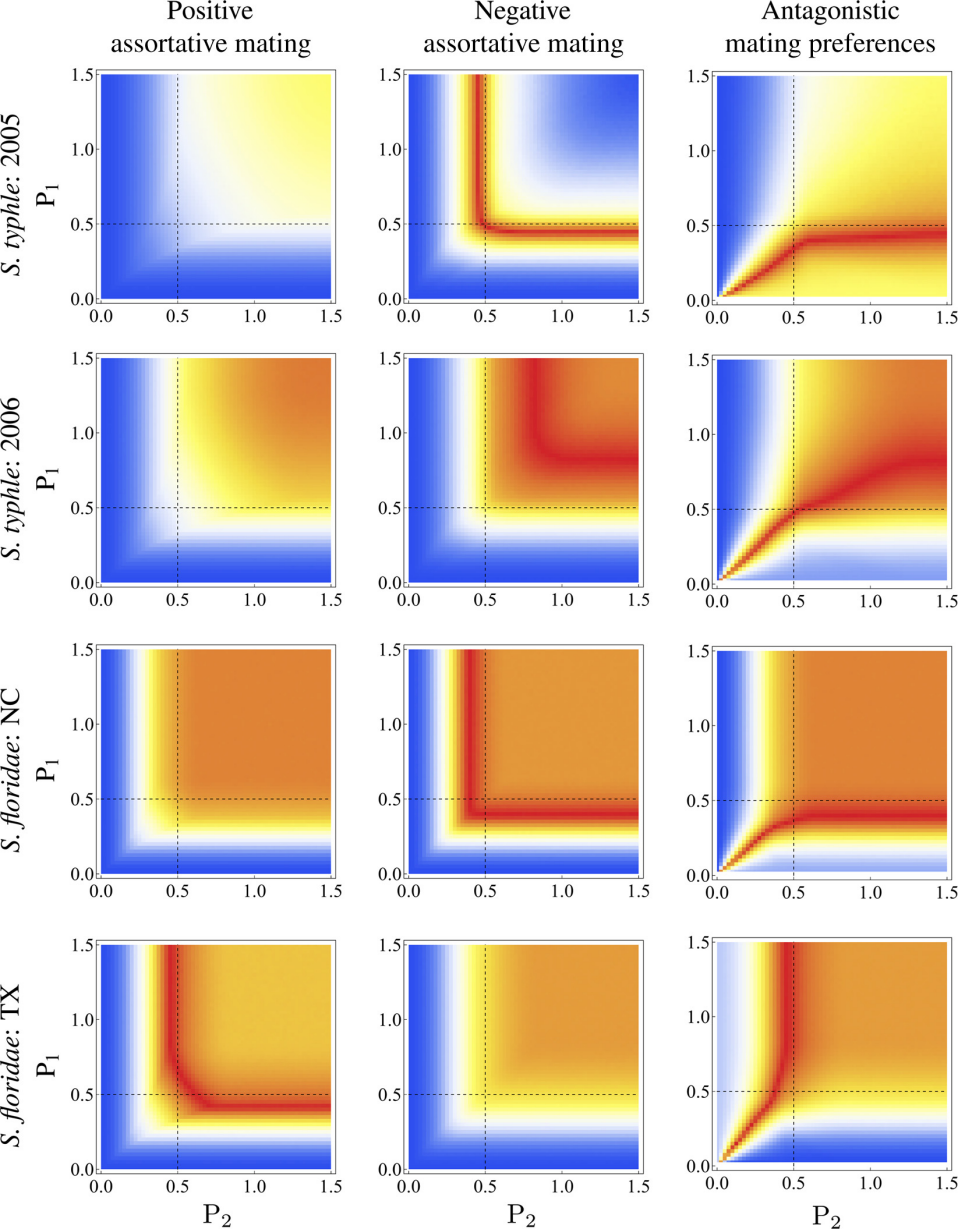
Results after each simulated population was statistically analyzed and compared with collections of *S. typhle* (2005 and 2006) and *S. floridae* (North Carolina and Texas). All pairing patterns that best describe the natural population are denoted in red. Texas is the only population described by the positive model. This is seen in two scenarios, the positive assortative mating and antagonistic mating preference where P_2_ (positive heuristic) preferences drive mate pairing patterns in the positive direction. This effect is reversed for *S. typhle* (2005 and 2006) and *S. floridae* (North Carolina) where the negative model best describes the relationship, and P_1_ (negative heuristic) preferences drive a negative pattern for the antagonistic mating preference model.

Simulations using only positive or negative assortative mating regimes showed no evidence for strong trait preferences among the different collections of *S. typhle* and *S. floridae* (Table 3, Fig. 1). Consistent with expectations, the comparative analysis between the natural and simulated populations revealed similar preferences for body size irrespective of sex because the mate with the stronger preference regime has the greatest influence on the resulting mating pattern. A positive size-assortative mating model best described the Texas *S. floridae* population while the negative size-assortative mating model best described the other three populations, *S. floridae* (North Carolina) and *S. typhle* (Gåsö 2005, 2006; Fig. 3). Based on comparisons of simulated regressions with regressions from each population, we extracted a best-fit model of P_1_ = 0.450, P_2_ = 0.650 (negative model) for *S. typhle* Gåsö 2005; P_1_ = 0.825, P_2_ = 1.175 (negative model) for *S. typhle* Gåsö 2006; P_1_ = 0.725, P_2_ = 0.400 (negative model) for *S. floridae* North Carolina population, and P_1_ = 0.850, P_2_ = 0.450 (positive model) for *S. floridae* hailing from the Texas population (Table 3, Fig. 3). Overall, our results showed that preferences have a weak effect on the pairing patterns found in the sampled populations of pipefish following strict positive or negative assortative mating.

**Table 3:** Best-fit models of the regression of males and females from field data based on simulated preferences. Number of matched mating pairs (*n*), slope, and intercept of field data are reported, and the model, number of simulated mate pairs (*n*), strength of preference for mate 1 and 2 (P_1_, P_2_), slope, intercept for simulation models, and the distance between field and simulation models based on slope and intercept estimates are reported for each sample of *Syngnathus typhle* and *Syngnathus floridae*. See text for model descriptions

	Field data				Model data					
		
	*n*	Slope	Intercept	Model	*n*	P_1_	P_2_	Slope	Intercept	Distance
*Syngnathus typhle*: 2005	18	−0.370615	283.752	Negative	100	0.450	0.650	−0.443323	284.026	0.0029767
				Antagonistic	100	0.425	1.075	−0.449761	283.710	0.0003165
*Syngnathus typhle*: 2006	28	−0.041894	213.172	Negative	100	0.825	1.175	−0.091180	213.192	0.0003087
				Antagonistic	100	0.825	1.425	−0.090570	213.136	0.0002837
*S. floridae*: NC	21	−0.146256	146.609	Negative	100	0.725	0.400	−0.112289	146.607	0.0029780
				Antagonistic	100	0.400	0.850	−0.111466	146.609	0.0002373
*S. floridae*: TX	28	0.165558	122.274	Positive	100	0.850	0.450	0.154709	122.141	0.0010898
				Antagonistic	100	0.800	0.450	0.155656	122.042	0.0013366

Only the positive and antagonistic models are reported for *S. floridae*: TX samples, all others report the negative and antagonistic models.

Simulations of antagonistic mating preferences, where both mates have strong preferences albeit in opposite directions, can result in a population without assortative mating if the strength of the preferences is similar in each sex. This is an interesting scenario, as it does not negate the existence of preferences, only that the resulting population will exhibit no size-assortative mating. Furthermore, simulations of antagonistic preferences clearly emphasized the directionality of mating pairs found in each population. Results show that the mate with the negative heuristic (P_1_) determines the outcome as observed for *S. typhle* Gåsö (2005) and *S. floridae* from the North Carolina population (Table 3, Fig. 3). On the other hand, the mate with the positive heuristic preference (P_2_) determines the outcome for *S. floridae* from the Texas population (Table 3, Fig. 3). Results of best-fit regression analyses showed similar values of preferences for antagonistic models as compared with the dominant preference of positive or negative models, demonstrating that the individual with the strongest preference determines the direction of assortative mating. Overall, whether observed mating patterns are due to mutual or antagonistic preferences, the strength of preferences is necessarily weak to generate patterns consistent with our models.

## Discussion

In this study, we investigate body size relationships of two polygynandrous species of pipefish in natural populations that have been sufficiently sampled to match a high proportion of pregnant males with their respective female partners. Despite the strong predilection to choose mates of larger size in at least one of the species (*S. typhle*) based on laboratory experiments (Berglund et al. 1986, 2005; Berglund 1994) and potentially males from the same Texas population of *S. floridae* (S. Scobell pers. comm. 2013), we do not find any convincing evidence that size-assortative mating takes place in the wild for either of these species. Rather, in three of four locations or times sampled, we see a potential trend toward negative size-assortative mating. Therefore, if positive assortative mating does exist in these species, it would probably have to be on a trait uncorrelated with body size. Our heuristic model also indicates that body size preferences in each of the populations studied herein fit nearly the full range of all breeding individuals. Thus, if the sexes display preferences for body size, then the manifestation of such preferences in the field appear to be weak, and mating pairs encompass all combinations of body size except the most extreme outliers in the population distribution.

Our results also provide evidence that two potentially important traits for reproductive success, order of mating and the number of eggs contributed by each female, do not affect the body size relationship between the sexes. On average, large females are not any more successful than small females in pairing with preferred larger males regardless of the fact that larger females may confer a higher fitness to offspring by either producing larger eggs or offspring with higher fitness (Berglund et al. 1986; Ahnesjö 1992). At the same time, larger males do not pair with preferred larger females in spite of the strong likelihood that male mate choice operates in these species due to sex-role reversal (Berglund et al. 1986). In these species, the first female to mate with a male contributes more eggs per clutch than subsequent females (Berglund et al. 1988; Partridge et al. 2009), and therefore, one can envisage a scenario in which males may be choosy for the first female and then less choosy for additional mate pairings. However, there is no evidence in our data set to support such a scenario, as our results indicate that order of mating does not influence body size pairing in either species.

Understanding how individual preferences lead to population patterns of assortative mating is a formidable task, particularly because various types of preferences such as strong mutual, directional, or antagonistic mate choice may result in similar patterns of assortative mating in nature (Burley 1983). Detailed information concerning how individual mating preferences may change due to the abundance of high-quality mates or may be modified, for example, by the body size of the individual, is simply unknown for most species. Furthermore, information concerning mating preferences generally comes from laboratory mate-choice trials, which may or may not reflect true preferences in nature. For these reasons, we developed a general simulation model based on simple heuristic rules to investigate how preferences for similar body size influence population pairing patterns. While the heuristics are admittedly simplified, our model offers a glimpse into the strength of preferences that may explain our actual patterns of mating in the wild.

The first major outcome of our simulation models is that the choosier sex determines the strength and direction of assortative mating patterns. Whether the preference of the second mate is strong or weak has little effect on the overall outcome of assortative mating patterns, an observation consistent with other reports of assortative mating models (Burley 1983; McNamara and Collins 1990; Härdling and Kokko 2005). The second major inference from model simulations is that the mating preferences are necessarily weak and tend to be negative in our natural populations under the strict positive and negative assortative mating models. The strength of the preferences under antagonistic mating patterns, on the other hand, does not show assortative mating when preferences are equal but can show variable strengths and directions of assortative mating depending on the choosier sex. However, antagonistic mating is an unlikely scenario in pipefish because both sexes prefer larger mates in laboratory-based preference trials. Regardless, if males and females have different reproductive goals, this situation represents another way that patterns of assortative mating may not occur in nature despite strong individual preferences, albeit in opposite directions.

How then can we reconcile the maintenance of a preference for body size with the absence of size-assortative mating in pipefishes? The answer to this problem does not appear straightforward. One potential explanation as to why individual preferences may not manifest into strong assortative mating is that mate choice in the wild is limited by various ecological constraints. Examples of ecological constraints to mate choice may include dietary considerations or energetic costs to gamete production (Hayward and Gillooly 2011). Strong competition for high-quality mates may also limit the amount of mating opportunities experienced at a given time, thereby driving up the costs to mate with preferred partners (Servedio and Lande 2006; South et al. 2012). At the community level, interspecies competition and predation (Berglund 1993; Fuller and Berglund 1996) may play a role in modifying mate-choice behaviors, but the extent to which these factors influence mate choice in the wild are largely unknown. Environmental conditions that affect mate choice may also play a role in mediating assortative mating. For example, environmental challenges to the perception distance and mate encounter rate may alter mate-choice decisions (Sundin et al. 2010; Candolin and Wong 2012). Here, we focus our attention on demographic processes such as local population density and breeding synchrony because these processes are likely linked to specific mate-choice behaviors, and data are available for these species.

Population density is likely to affect mate choice by moderating both encounter rates of potential mates as well as competitors (Kokko and Rankin 2006). For example, in populations with low density, few mate encounters may make individuals less choosy and consequently mate with the first available partner. In the opposite extreme, the presence of many potential partners and competitors may similarly prevent individuals from choosing optimally in high-density situations (McLain 1992; Mills and Reynolds 2003; Pomfret and Knell 2008). This latter possibility may be the most likely scenario in *S. typhle*. Because of its northerly distribution, *S. typhle* has a more restricted breeding season from May to August (Vincent et al. 1994, 1995) and population densities in shallow seagrass beds peak during the mating season (Vincent et al. 1995). Competition may also be reduced at the beginning of the mating season when many females and males become simultaneously receptive. In contrast, pregnant male *S. floridae* have been found in nearly all months of the year with a peak in the breeding season between July and August (Brown 1972; Mercer 1973). Compared with *S. typhle*, *S. floridae* has a shorter gestation period (7–14 days compared with 20–40 days), a more asynchronous receptivity to mating and a smaller local population size suggesting that competition, and thus mate choice, should be stronger within this species.

A second potential explanation as to why preferences may not result in assortative mating may lie in the intrinsic properties of the mating system of these two species. In monogamous species, an individual's reproductive success is dependent on the fecundity of its partner, and therefore, both partners should be choosy (Griffith et al. 2011). In support of this hypothesis, close relatives of pipefishes, the seahorses (Genus *Hippocampus*), show strong assortative mating patterns (Jones et al. 2003). In contrast, both species of pipefishes have a polygynandrous genetic mating system that is characterized by multiple mating in both males and females (Jones and Avise 1997b; Jones et al. 1999). It is interesting to note that the one population that shows a trend toward positive size-assortative mating (TX) also is the one in which males have the fewest matings per pregnancy on average. Thus, in polygynandrous species, individual preferences may exist but may not be as important in the wild as they appear to be from laboratory studies simply because individuals must relax their preferences somewhat to obtain a large number of mates while dealing with the ecological and demographic constraints imposed upon natural populations.

It is important to point out that while assortative mating can influence the intensity of sexual selection, the two processes may be unrelated to each other. For example, several studies demonstrate positive size-assortative mating at the population level without evidence for individual-level preferences (Taborsky et al. 2009; Thünken et al. 2012). These observations indicate that positive size-assortative mating can arise from the exclusion of some individuals from mating due to morphological or other preclusive reasons such as the inability to maintain a territory. Alternatively, sexual selection without size-assortative mating is also possible. An interesting case is the dance fly, *Rhamphomyia longicauda*, where males and females display extreme sexual dimorphism but show no size-assortative mating (Bussiere et al. 2008). In the case of pipefish, both species in this study demonstrate significant sexual selection on male and female body size due to higher mating and reproductive success of larger individuals (Jones et al. 1999, 2005; Mobley 2007). Moreover, despite the proposed ubiquity of assortative mating, there are a few examples where traits that seem to confer a fitness advantage do not appear to affect pairing patterns. For example, certain species of birds do not show assortative mating based on ornamental traits despite the maintenance of such traits in both sexes (Murphy 2008; van Rooij and Griffith 2012). Here, other forms of selection such as natural selection or social selection may help to explain the maintenance of mutual ornamentation in the absence of immediate benefits to mating or reproductive success (Tarvin and Murphy 2012; Tobias et al. 2012).

In summary, size-assortative mating in species with mutual preferences for body size, although oftentimes assumed, is not a foregone conclusion. Ecological constraints and the frequency of multiple mating may play important roles in preventing individual preferences from being fully realized into strong patterns of assortative mating in nature. Our results join a growing body of studies that find little or no support for assortative mating in wild populations where traditional paradigms, usually based on laboratory mate-choice experiments, suggest the opposite (Murphy 2008; van Rooij and Griffith 2012). Therefore, our findings underscore the need to quantify sexual selection and mate choice under both laboratory and field conditions to best understand how sexual selection and mate choice influence mating behavior in nature.

## Supporting Information

**Data S1.** Supporting information.

**Data S2.** Interactive heuristic model.
