# Supplementary material for: No evidence for size-assortative mating in the wild despite mutual mate choice in sex-role-reversed pipefishes
Source: Ecol Evol. 2013 Dec 11;4(1):67–78. doi: 10.1002/ece3.907 (PMC3894889; doi:10.1002/ece3.907)
Supplement: Supplementary file 1 [file ece30004-0067-SD1.docx]

**Supporting Information**

1. **Interactive heuristic model (.cdf format)**

The model was constructed using Mathematica software and can be viewed with the free Wolfram CDF Player (<http://www.wolfram.com/cdf-player/>).

1. **Algorithm for heuristic model (Mathematica v 8.0)**

Herein we describe the algorithm behind the simulations conducted using Mathematica v 8.0 (Wolfram Research Inc., Champaign, USA). We performed the simulations using two heuristics: T_i_ ± T_i_ *P and T_min_+(T_max_-T_i_ )± T_i_ *P. These were implemented into a routine function (modelPreference) from which a population, with a T_min_ and T_max_ and a preference pair (P_1_, P_2_) for males and females is created. The preference pair (P_1_, P_2_) was chosen from a real number distribution ranging from 0 to 1.5 in increments of +/- 0.025.

*modelPreference*=.

modelPreference[*T1*_max__*, T2*_max__, *T1*_min_*, T2_min_* _,preferenceOPt_,data_,popSize_]:=Module[{},

popT=Tuples[{Range[minV1,maxV1,1],Range[minV2,maxV2,1]}];

If[preferenceOPt==1,

t9=((minV2+(maxV2-#[[2]])-t2*#[[2]])<= #[[1]])&&((minV2+(maxV2-#[[2]])+t2*#[[2]])>= #[[1]])&&((minV1+(maxV1- #[[1]])-t1* #[[1]])<=#[[2]])&&((minV1+(maxV1- #[[1]])+t1* #[[1]])>= #[[2]])&

,

t9=(((#[[1]]-t1*#[[1]])<=#[[2]])&&((#[[1]]+t1*#[[1]])>= #[[2]])&&((#[[2]]-t2*#[[2]])<=#[[1]])&&((#[[2]]+t2*#[[2]])>= #[[1]]))& ];

Flatten[N[Table[Join[{t1,t2},sampleStats[Select[popT,t9],data,popSize,100]],{t1,0,1.5,0.025},{t2,0,1.5,0.025}]],1]

]

Each randomly extracted population was statistically analyzed using a linear regression model by calculating the regression coefficient (slope), and y-intercept. A regression function (regStats) was implemented for data sets with many y and one x. Random sampling of 10,000 populations was conducted using the sampling function (sampleStats).

*regStats =.*

regStats[popD_] := Module[

{ni, grp, ximeanyi, SSwithin, meanxy, meanmeanxy, squarexy, totalxy, regcoef, yint, explainedSumofSquares, unexplainedSumofSquares, MSamong, MSwithin, MSlinear, Fs},

ni = Count[First /@ popD, #] & /@ Union[First /@ popD];

grp = SplitBy[Sort[popD], First];

ximeanyi = Mean /@ SplitBy[Sort[popD], First];

SSwithin = Total[Flatten[(#[[2]] - #[[1]])^2 & /@ Transpose[{Last /@ ximeanyi, Last /@ # & /@ grp}]]];

meanxy = Mean[popD];

meanmeanxy = Mean[ximeanyi];

squarexy = Total[{#[[3]]*(#[[1]] - meanxy[[1]])^2, #[[3]] (#[[2]] - meanxy[[2]])^2} & /@ Flatten /@ Transpose[{ximeanyi, ni}]];

totalxy = Total[#[[3]]*(#[[1]] - meanxy[[1]]) (#[[2]] - meanxy[[2]]) & /@ Flatten /@ Transpose[{ximeanyi, ni}]];

regcoef = totalxy/squarexy[[1]];

yint = meanxy[[2]] - (regcoef*meanxy[[1]]);

regcoef, yint, Mean[ni]}

]

*sampleStats* =.

sampleStats[popT1_, rObserved_, iterate_, sampleSize_] := Module[

{popR2, d, pValue1, pValue2},

popR2 = RandomChoice[popT1, {iterate, sampleSize}];

d = regStats[#] & /@ popR2;

pValue1 = Length[Select[First /@ d, # >= rObserved[[1]] &]]/iterate;

pValue2 = Length[Select[First /@ d, # <= rObserved[[1]] &]]/iterate;

Join[{pValue1, pValue2}, Mean[d], rObserved]

]

The algorithm is incorporated using the following Export functions that create an output file for all possible pairing patterns and both heuristic models.

Export["datalist-"<>ToString[popSize]<>"-p11-"<>ToString[N[#[[2]]]]<>".dat",

modelPreference[Max[{#[[1,1]],#[[1,2]]}],Max[{#[[1,1]],#[[1,2]]}],Min[{#[[1,3]],#[[1,4]]}],Min[{#[[1,3]],#[[1,4]]}],1,#[[2]],popSize]

]&/@Take[Transpose[{maxmind2,regData}],4]

Export["datalist-"<>ToString[popSize]<>"-p21"<>ToString[N[#[[2]]]]<>".dat",

modelPreference[Max[{#[[1,1]],#[[1,2]]}],Max[{#[[1,1]],#[[1,2]]}],Min[{#[[1,3]],#[[1,4]]}],Min[{#[[1,3]],#[[1,4]]}],2,#[[2]],popSize]

]&/@Take[Transpose[{maxmind2,regData}],4]
